# Buckwheat Rutin Inhibits AngII-induced Cardiomyocyte Hypertrophy via Blockade of CaN-dependent Signal Pathway 

**Published:** 2014

**Authors:** Jin-xiu Chu, Guang-min Li, Xiu-juan Gao, Jian-xing Wang, Shu-ying Han

**Affiliations:** a *Department of Pharmacology,College of Elementary Medicine, Hebei United University, Tangshan 063000, PR China. *; b *Tangshan Women and Children’s Hospital Affiliated to Hebei United University, Tangshan 063000, PR China.*; c *Department of Traditional Chinese Medicine, Hebei United University, Tangshan 063000, PR China.*; d *Jitang College of Hebei United University, Tangshan 063000, PR China.*

**Keywords:** Rutin, Buckwheat, Angiotensin II, Cardiac hypertrophy, Calcineurin

## Abstract

Buckwheat rutin has been found to be able to inhibit angiotensin II (AngII) - induced hypertrophy in cultured neonatal rat cardiomyocytes, but the mechanism remains uncertain. In this study, myocardial hypertrophy model was made by adding AngII to the medium of cardiac myocytes of neonatal rats; meanwhile, different concentrations of buckwheat rutin were applied to observe their effects. Intracellular Ca^2+^ level was detected by Hitachi - 850 fluorospectrophotometer, calcineurin (CaN) activity was measured by colorimetric method, the expression of CaN protein was observed with immunocytochemistry, and the proto - oncogene c - fos mRNA expression was assessed with reverse transcription polymerase chain reaction (RT - PCR). Compared with control group, AngII could greatly stimulate the increase of intracellular Ca^2+^ level, the activities and protein expression of cardiomyocytes CaN, and the expression of proto - oncogene c - fos mRNA in cultured neonatal rat cardiomyocytes, which could be effectively decreased by buckwheat rutin. Our results demonstrated that buckwheat rutin exhibited inhibitory effect on AngII - induced hypertrophy in cultured neonatal rat cardiomyocytes via Ca^2+^ antagonism action thus block the CaN - dependent signal pathway.

## Introduction

Myocardial hypertrophy, which often leads to congestive heart failure, severe arrhythmia and disruption of important artery branches, is a commonly seen complication of cardiovascular system diseases. It is an independent risk factor causing the increase of death rate of cardiovascular diseases, and it gravely threatens the physical and mental health and life quality of human being. 

Myocardial hypertrophy is commonly caused by hypertension, vascular disease of the heart, coronary heart disease, myocardial infarction and congenital heart disease *etc.* In recent years, the experts have thought highly of the imbalance of neurohumor factors such as catecholamine, endothelin-1, various growth factors, especially the renin-angiotensin-aldosterone system that causes myocardial hypertrophy. Generous investigations have manifested that AngII has growth factor like effect. It can cause the increase in total protein content of cardiac myocytes and in protein synthesis rate, which results in the hypertrophy of cardiac myocytes. It can also increase the synthesis and excretion of matrixes of myocardium interstitial cells (mainly cardiac fibroblasts) and causes myocardial hypertrophy or myocardial fibrosis. The elevation of intracellular Ca^2+^ level is indicated to be the central link of hemodynamics overload and various neurohumor factors inducing myocardial hypertrophy. Ca^2+^ - calmodulin (CaM) dependent CaN pathway is a recently discovered important signal transduction pathway, which directly participates in several extracellular signal pathways that cause myocardial hypertrophy.

As to the treatment of myocardial hypertrophy, immunodepressant Cyclosporin A (CsA) is studied the most. CsA could effectively suppress the expression of myocardial CaN-NFAT_3_-GATA_4_ thus inhibit myocardial hypertrophy and improve congestive heart failure. Due to the impact of side effects, CsA has not been spreadly applied in the clinic.

Rutin, also named as globulariacitrin and rutoside, possess many pharmacological activities including lowering the capillary permeability, anti-inflammatory, anti-anaphylaxis, anti-tumor, anti-bacteria, anti-virus and hepato-protection. Buckwheat rutin is an active ingredient extracted from *Fagopyrum esculentum* Moench. [F. sagittatum Gilib; Polygonum fagopyrum L.] with abundant resources and mature techniques. Our previous studies have found rutin could inhibit AngII - induced hypertrophy in cultured neonatal rat cardiomyocytes. To investigate the mechanism, intracellular Ca^2+^ level, the activities and protein expression of CaN, and the expression of proto - oncogene c - fos mRNA in cultured neonatal rat cardiomyocytes induced by AngII were determined. The finding could provide experimental evidence for the development of new drugs for prevention and cure of myocardial hypertrophy.

## Experimental

*Plant materials and isolation of rutin*

Buckwheat, flowers and leaves collected in late autumn from Ku Lun, Inner Mongolia (China), was identified by the Department of Natural Medicinal Chemistry, Hebei United University, Tangshan, China. A voucher specimen is deposited in the Department of Pharmacology, Hebei United University, Tangshan, China. Buckwheat rutin was isolated with the method demonstrated in the literature (1).

*Cell culture and induction of hypertrophy*

Primary cardiac myocytes were prepared from 1 to 3-day-old Wistar rats hearts following the sequential enzymatic digestion method described previously and were cultured in Dulbecco’s Modified Eagle Medium (DMEM) supplemented with 10% FBS, 100 U/mL penicillin, and 100 μg/mL streptomycin at 37 °C in a 5% CO_2_ humidified incubator. Hypertrophy of cardiac myocytes was induced by adding 10^-7 ^mol/L AngII, a known promoting growth factor, to serum-free medium (1). All procedures were approved by the Ethical Committee for Laboratory Animals of Hebei United University and were performed in accordance with the Principles in Animal Care and Use of the Committee.

*Experimental groups(1)*

The primarily cultured cardiac myocytes were synchronized with serum-free DMEM for 24 h as they grew to the proper density, then the cells were grouped as follows:

Control group: Cultured in serum-free DMEM.

Model group: Cultured in serum-free DMEM supplemented with 10^-7 ^mol/L Ang II.

Rutin I group: Cultured in serum-free DMEM supplemented with 0.8 mg/L rutin 30 min prior to Ang II.

Rutin II group: Cultured in serum-free DMEM supplemented with 4.0 mg/L rutin 30 min prior to Ang II.

Rutin III group: Cultured in serum-free DMEM supplemented with 8.0 mg/L rutin 30 min prior to Ang II.

Different dosages of rutin were chosen according to previous studies.

*Detection of intracellular Ca* ^2+^ *level* (2)

Cardiac myocytes were treated with various concentrations of rutin for 24 h, then, intracellular Ca^2+^ levels were detected with Fura-2/AM, the specific indicator of Ca^2+^. Briefly, cardiac myocytes were loaded with 4 µmol/L Fura-2 / AM (dissolved in dimethyl sulfoxide with 0.02% pluronic) for 30 minutes at 37 °C in a humidified incubator with 95% air /5% CO_2_. Cells were then washed three times with modified Hanks' buffer containing (mmol/L) NaCl 137, NaHCO_3_ 4.2, NaHPO_4_ 3, KCl 5.4, KH_2_PO_4_ 0.4, CaCl_2_ 1.3, MgCl_2_ 0.5, MgSO_4_ 0.8, glucose 10, and HEPES 5 (pH 7.4). Fluorescence was determined with the Hitachi - 850 fluorospectrophotometer using dual excitatory wavelengths of 343 and 380 nm and a single-emission wavelength of 520 nm. [Ca^2+^]_i_ was determined with the equation of Grynkiewicz et al: [Ca^2+^]_i_=*K*_d_xß(R-R_min_)/(R_max_-R), where *K*_d_ is the dissociation constant for fura 2 - Ca^2+^ and taken to be 224 nmol/L, ß is the ratio of fluorescence at 380 nm and zero Ca^2+^ (F_380min_) and saturating Ca^2+^ (F_380max_) conditions, and R is the ratio of fluorescence obtained with excitation at 343 and 380 nm, with min and max subscripts denoting the ratios obtained under Ca^2+^-free and Ca^2+^-saturated conditions, respectively. The unit of [Ca^2+^]_i _is nmol/ L. The test was repeated for six times. 

*Preparation of c* *ardiac myocytes* *and measurement of protein content*

 Cardiac myocytes were dealed with drugs and AngII for 24 h, and then they were washed thoroughly with PBS for three times and collected, precooled homogenate (50 mmol/L Tris PH7.5, 0.1 mmol/L EGTA, 1 mmol/L EDTA, 0.5 mmol/L DTT, 50 μg/mL PMSF, 50 μg/mL STI, 5 μg/mL leupeptin, 5 μg/mL aprotinin) was added and the cells were broken into pieces by repeated freezing and thawing, and centrifuged at 4 °C, 12000 g for 10 min. The supernatant was obtained and protein content was measured by Brad-ford method. 

*Measurement of* *CaN activity*

CaN activity was measured by colorimetric method referred to the literature (3) with some modification. Briefly, after 20 μL of above supernatant and 380 μL of substrate reacted at 30 °C for 10 min, 0.5 mmol/L NaCO_3 _and 0.4 mmol/L EGTA were added to stop the reaction and the absorbance was measured by automatic microplate reader at 410 nm (results expressed as A_410nm_). The substrates were used for zero setting. Substrate I contained 50 mmol/L Tris-HCl PH 7.4, 0.5 mmol/L DTT, 0.2 mg/mL BSA, 10 mmol/L PNPP, 0.5mmol/L MnCl_2_, 0.2 mmol/L CaCl_2_, 0.3 μmol/L CaM. Substrate II contained 3 mmol/L EGTA without CaCl_2_ and CaM. A_410nm_ with substrate I reflected the activity of total phosphatases, and A_410nm_ with substrate II reflected the activity of other phosphatases except CaN. So, A_410nm_ of CaN was their subtraction. CaN activity was expressed as A_410nm_/mgpr.

*CaN protein expression observed by immunocytochemistry*

Primarily cultured cardiac myocytes were seeded in 24-well plate with 10 mm×8 mm sterile coverslips (3×10^5 ^cells/mL, 1 mL per hole) and cultured for 48 h prior to 24 h’ synchronization, dealed with drugs for another 24 h before termination, fixed with 4% paraformaldehyde. CaN protein expression was detected using mouse monoclonal antibody raised against rat CaN-α (Wuhan Boster Bio - Engineering Limited Company, China) as first antibody and biotin-labeled goat anti-mouse IgG as second antibody. Photos were analyzed by MoticMed System 6.0A (Beihang University, Beijing, China).

*Measurement of c-fos mRNA by RT-PCR*

Total RNA was isolated by Trizol and its density and purity were monitored by extra-violet spectro- luminosity instrument. The ratio of A260 /A280 was among 1.8~2.0. Apex time of c-fos mRNA expression stimulated by Ang II was observed and the effect of buckwheat rutin on c-fos mRNA expression at apex time was evaluated by RT-PCR. The following c-fos primers designed by Stepien (4) were used: sense primer 5'-AGC TGA CAG ATA CGC TCC AA-3', and antisense primer 5'-TAG GTG AAG ACA AAG GAA GAC G-3', length of the amplification fragment was 556 bp. The control GAPDH primer: sense primer 5'-TGC TGA GTA TGT CGT GGA G-3', and antisense primer 5'-GTC TTC TGA GTG GCA GTG AT-3'), length of the amplification fragment was 288 bp (Synthesized by Shanghai biological engineering limited company). Destination gene magnification was carried out according to directions of RT-PCR kit. The conditions were as follows: synthesis and pre-denaturation of c-DNA at 37 °C for 60min, one cycle at 95 °C for 5min, amplification of PCR by 35 cycles at 94 °C for 50 s, 60 °C for 45 s and 72 °C for 1 min, and then extension at 72 °C for 7 min. Amplified product of c-fos and GAPDH 5 μL plus 2 μL sample buffer solution were electrophoresed for 40 min in 2% agarose gel at 80V respectively. The electrophoretogram was scanned by gel image analysis instrument and gray scale measurement was carried out through QuantityOne image analysis software. The ratio of c-fos to GAPDH was calculated.

*Statistical analysis*

Statistical analyses were performed with the GraphPad instat software. Data were expressed as mean ± standard deviation (S.D). Statistical significances were analyzed using the ANOVA test. A value of *P *< 0.05 was considered significant. 

## Results and Discussion

Results showed [Ca^2+^]_i_ in control group, model group, Rutin I group, Rutin II group and Rutin III group were 121 ± 22 nmol/ L, 178 ± 25 nmol/ L (*P *< 0.01 v.s. control group ), 153 ± 27 nmol/ L, 132 ± 21 nmol/ L (*P *< 0.05 v.s. model group ), 128 ± 24 nmol/ L (*P *< 0.05 v.s. model group ) respectively. Buckwheat rutin inhibited increase of [Ca^2+^]_i_ in Ang II - induced cardiac myocytes remarkably.

**Figure 1:**
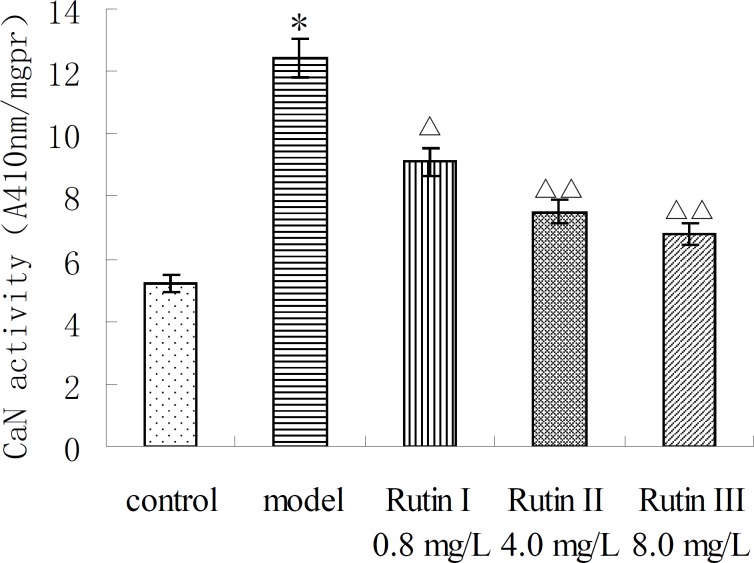
CaN activity of AngⅡ-induced cardiac myocytes treated with buckwheat rutin. ^*^P < 0.05 vs control group; ^△^ *P* < 0.05 , ^△△^ *P* < 0.01 vs model group (n＝8).

**Figure 2:**
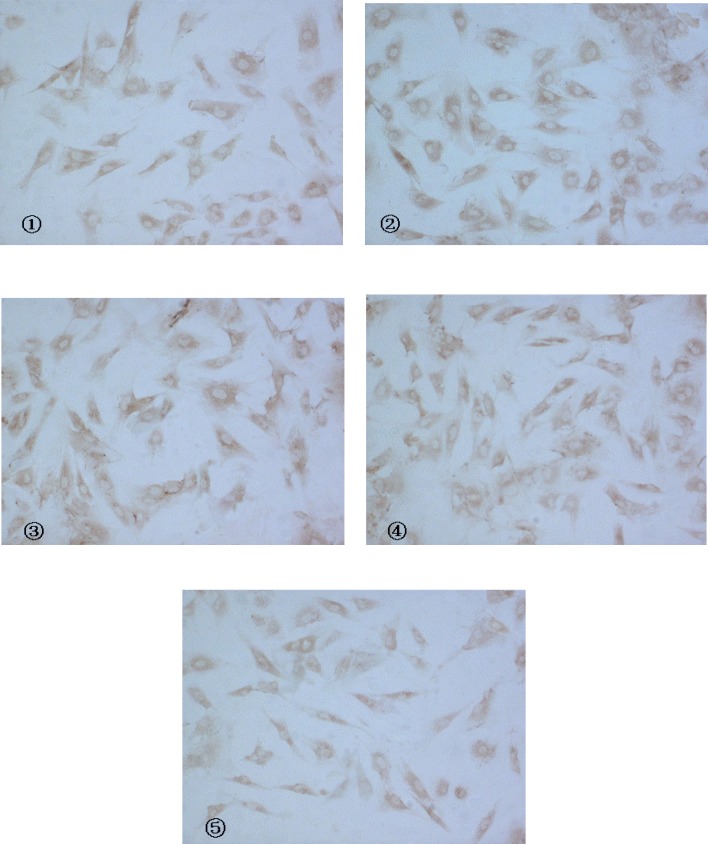
Expression of CaN protein in cardiac myocytes with CnA anti-body as a marker (IC, × 200) (① control ②model ③Rutin I 0.8 mg/L ④Rutin II 4.0 mg/L ⑤Rutin III 8.0 mg/L).

**Figure 3:**
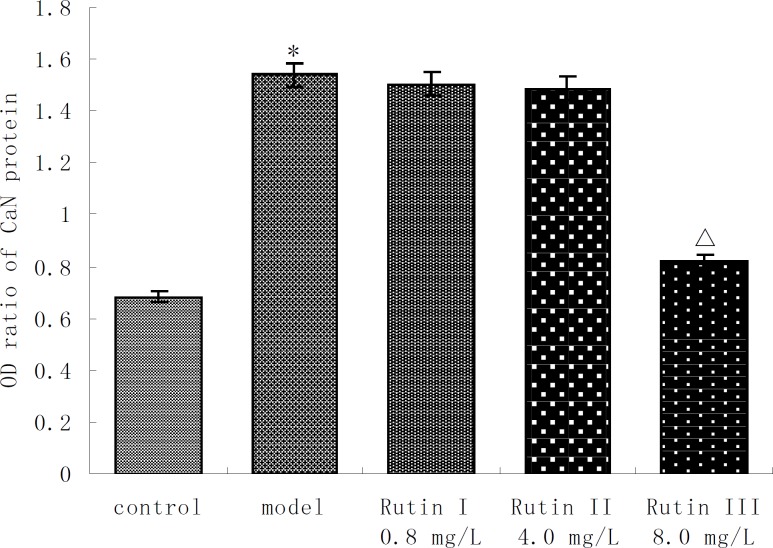
Expression of CaN protein in AngⅡ-induced cardiac myocytes treated with buckwheat rutin (OD ratio , n＝6 ). ^*^P < 0.01 *vs* control group; ^△^*P* < 0.01 *vs* model group

CaN activity of 10^-7 ^mol/L Ang II -induced cardiac myocytes was twice more than that of control group. Buckwheat rutin at 0.8 mg/L, 4.0 mg/L, 8.0 mg/L could obviously inhibit the increasing of CaN activity caused by Ang II (*P *< 0.05, *P *< 0.01, *P *< 0.01) and showed dose-dependent relationship (Figure 1). Results of immunocytochemistry showed that in control group, CaN protein mainly presented intracytoplasm in cardiac myocytes especially remarkable around the nuclear membrane, *i.e*., cytoplasm of cardiac myocytes was brown while the nuclei were almost not stained. As shown in the photos (Figure 2), the brown granulas in the cytoplasm of cardiac myocytes stimulated by Ang II accumulated obviously especially remarkable around the nuclear membrane. The staining in buckwheat rutin treated groups was much lighter than the model group. When analyzed by the MoticMed System 6.0A, the CaN protein expression (OD ratio) in cardiac myocytes of Ang II group (1.541 ± 0.075) was notably increased compared with the control group (0.682 ± 0.038) (*P*<0.01), whereas, buckwheat rutin at dosages of 0.8 mg/L, 4.0 mg/L, 8.0 mg/L could greatly decrease the Ang II induced expression of CaN protein to 1.503 ± 0.064, 1.488 ± 0.071 and 0.822 ± 0.051 respectively (*P*<0.01) in a dose-effect relationship (Figure 3). 

**Figure 4:**
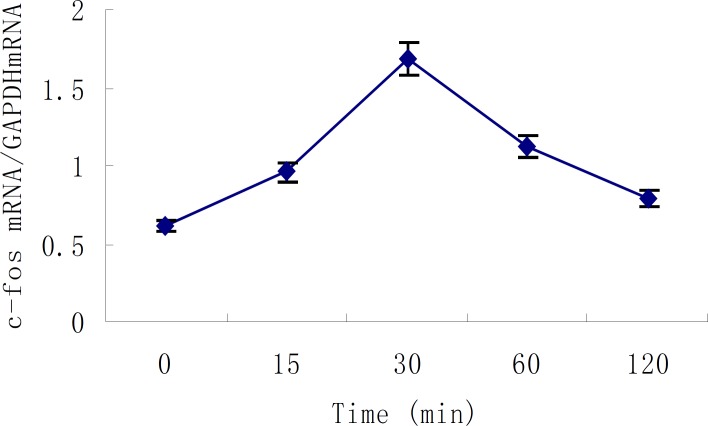
Time course of Ang II - induced c-fos mRNA expression in cultured neonatal rat cardiac myocytes (mean ± SD, n=5).

**Figure 5:**
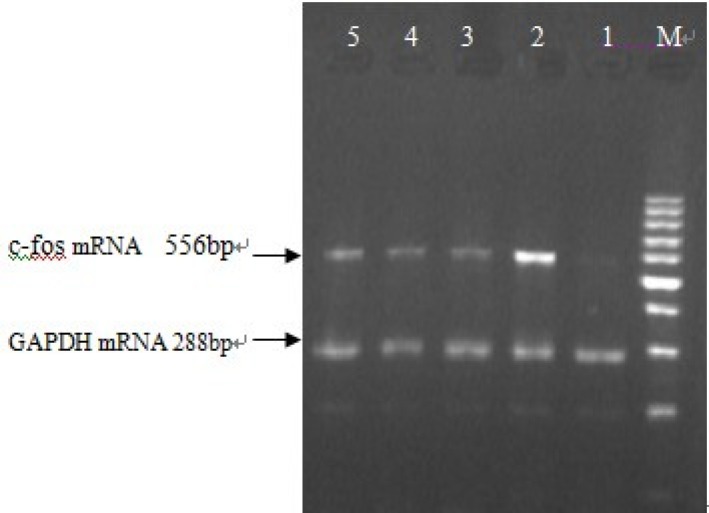
Effect of buckwheat rutin on c-fos mRNA expression in cardiac myocytes of neonatal rats induced by Ang II (M: Marker, 1: Control group, 2: Model group, 3: Rutin I 0.8 mg/L, 4: Rutin II 4.0 mg/L, 5: Rutin III 8.0 mg/L).

Apex time of c-fos was measured, and the results of five repeated experiments indicated that c-fos mRNA expression started to increase 15min after the action of 10^-7 ^mol/L Ang II in cultured neonatal rat cardiac myocytes, and reached its peak at 30min which was 2.8 times that of control group (*P *<0.01). After that, the expression of c-fos mRNA began to decrease gradually and nearly went back to normal level in 2 hours. This demonstrated that c-fos mRNA expression was stimulated by Ang II and existed specific time difference (Figure 4). The primarily cultured cardiac myocytes were synchronized with serum-free DMEM for 24 h as they grew to the proper density, then they were treated with different dosages of buckwheat rutin at 30 min prior to Ang II. At the apex time when the expression of c-fos mRNA reached the peak, *i.e*., 30 min after the addition of Ang II, total RNA was isolated from the cells and the expression of c-fos mRNA was detected. Results showed that c-fos mRNA expression in Ang II group was much higher than that in control group (*P *< 0.01), while different concentrations of buckwheat rutin could obviously reverse these changes. Compared with Ang II group, c-fos mRNA expression in different concentrations of buckwheat rutin decreased 26% (*P *< 0.05), 36% (*P *< 0.01) and 47% (*P *< 0.01) individually (Table 1, Figure 5). 

**Table 1:** Effects of buckwheat rutin on c-fos mRNA expression in cardiac myocytes of neonatal rats induced by Ang II.

**Group **	**Dose**	**c-fos mRNA / GAPDH mRNA** **(** **mean ± SD** **)**
Control	-	0.69 ± 0.22
Model	10^-7 ^mol/L Ang II	1.61 ± 0.35*
Rutin I	10^-7 ^mol/L Ang II + 0.8 mg/L rutin	1.19 ± 0.44△
Rutin II	10^-7 ^mol/L Ang II + 4.0 mg/L rutin	1.03 ± 0.24△
Rutin III	10^-7 ^mol/L Ang II + 8.0 mg/L rutin	0.86 ± 0.22△

* *P *< 0.01 *vs* control group;

△ *P *< 0.01 *vs* model group (n=10).

Myocardial hypertrophy and fibrosis are the basic responses of myocardium to the alteration of mechanical load and neurohumor factors. The pathological changes of myocardial hypertrophy mainly include hypertrophy of cardiac myocytes and proliferation of non- cardiac myocytes. Renin-angiotensin system (RAS) is one of the most important reasons besides pressure overloading, nordrenaline and thyrine for myocardial hypertrophy. Ang II, the chief active peptide of RAS, is an effective promoter for cell growth which can facilitate the growth of various cardiovascular cells such as the cardiac myocytes, cardiac fibroblasts and vascular smooth muscle cells (5, 6). In recent years, the role of Ang II in the mechanism of myocardial hypertrophy has been investigated intensively and researchers have found that Ang II acts on AT_1_ receptor to promote cell growth in a concentration-dependent manner (7). Ang II receptors have two major subtypes, AT_1_ and AT_2 _(8, 9), and the former is considered to be present on the surface of human and rat cardiac myocytes with high affinity. Ang II can activate phospholipase C β by stimulating its G - protein coupled receptor (GPCR) subtype G_q/11_, thus leads to the production of the second messenger diacylglycerol (DAG) and inositol (1,4,5)-trisphosphate (IP_3_). DAG can activate PKC, as a result, the cell membrane ion channel Ca-_L _opens and Ca^2+^ inflows, meanwhile, PKC can act on the down level protein substrates and promote myocardial hypertrophy. IP_3 _combines directly with its receptors in sarcoplasmic reticulums and endoplasmic reticulums and causes the release of Ca^2+ ^stored in cellular nuclear envelope gaps to caryoplasms and cytoplasms (10-12). Moreover, AT_2_ was also reported to play a functional role in the cardiac hypertrophic process *in-vivo* (13).

Viewed from cellular and molecular levels, myocardial hypertrophy consists of the following three fundamental aspects, *i.e*., extracellular stimulation, intracellular signal transduction and intranuclear activation and rearrangement of genes, which evoke the cells to hypertrophy phenotype changes. Different stimuli of hypertrophy can cause activation of specific genes and form various types of distinctive myocardial hypertrophy (14), which depends mainly on the signal pathway they trigger. At present, three signal pathways are considered important in inducing myocardial hypertrophy and fibrosis (15), *i.e*., PKC cascade, MAPK cascade and CaN pathway. CaN is belonged to serine/threonine protein phosphatase, also called protein phosphatase 2 B (PP2B), which is the unique protein phosphatase found to be activated by Ca^2+^ and calmodulin (CaM). CaN is a heterodimer composed of one 61 KD catalytic subunit CnA and one 19 KD regulatory subunit CnB, and generally distributes in a variety of tissues including the heart. It was reported that all the increase of intracellular Ca^2+ ^from different origins could activate CaN. The nuclear factors of activated T cells (NFAT_3_) could be dephosphorylated by activated CaN, enter the cell nuclei, and act on the zinc finger transcription factor GATA_4_ to cause hypertrophy of cardiac myocytes and expression of embryo genes (16-19). Hypertrophy could be suppressed in transgenic mice expressing excessive CaN when given specific inhibitor cyclosporin A to them after birth (20). This illustrated that CaN signal transduction exerted significant role in myocardial hypertrophy process. Furthermore, it was reported that CaN dependent signal pathway participated in Ang II - induced hypertrophy of cardiomyocytes and proliferation of cardiac fibroblasts (21-23). Proto-oncogene c-fos is one of the earlier period respond genes, the expression of which could be induced by many stimuli causing myocardial hypertrophy such as Ang II, endothelin, heart pressure overloading by coarctation of abdominal aorta, and dragging of cultured myocardial cells. The excessively expressed c-fos is an important intranuclear transcription factor in the development of myocardial hypertrophy.

Buckwheat rutin is a major component of buckwheat flavonoids, and rutin exists in no grains other than buckwheat. Previous studies (1, 24) have found that buckwheat rutin could inhibit the hypertrophy of neonatal rat cardiac myocytes induced by Ang II. Our results showed that buckwheat rutin could inhibit the increasing of above parameters in Ang II -induced cardiac myocytes, which manifested that buckwheat could inhibit hypertrophy of Ang II -induced neonatal cardiac myocytes by blockade of CaN - dependent signal pathway. This provided potent experimental basis for the development of drugs preventing and treating cardiac hypertrophy. As it should be, the signal transduction pathways inside cardiac myocytes are rather complex, so the mechanism of buckwheat rutin need further investigation.
